# Sports Injuries in Wheelchair Rugby – A Pilot Study

**DOI:** 10.1515/hukin-2015-0098

**Published:** 2015-01-12

**Authors:** Joanna Bauerfeind, Magdalena Koper, Jacek Wieczorek, Piotr Urbański, Tomasz Tasiemski

**Affiliations:** 1 Department of Sport for People with Disabilities, University School of Physical Education Poznań, Poland

**Keywords:** tetraplegia, team sports, disability

## Abstract

The aim of the study was to analyze etiology and the incidence of sports injuries among wheelchair rugby players. Moreover, we verified if the levels of aggressiveness and anger presented by the athletes and their roles in the team influenced the incidence and severity of the injuries. The study involved 14 male players, members of the Polish National Wheelchair Rugby Team. During a 9-month period, the athletes participated in up to 9 training camps and 4 Wheelchair Rugby tournaments. The study was based on the Competitive Aggressiveness and Anger Scale, registry of sports injuries consulted and non-consulted with a physician and a demographic questionnaire. The following observations were made during the 9-month period corresponding to a mean of 25 training and tournament days: 1) wheelchair rugby players experienced primarily minor injuries (n=102) that did not require a medical intervention, 2) only four injuries needed to be consulted by a physician, 3) sports injuries occurred more frequently among offensive players than in defensive players, 4) offensive players showed a tendency to higher levels of anger and aggressiveness than defensive players. It can be concluded that wheelchair rugby is a discipline associated with a high incidence of minor injuries that do not require a medical intervention. The incidence rate of injuries during the analyzed period was 0.3 per athlete per training day.

## Introduction

Spinal cord injuries (SCI) involving the cervical segment of the spine (tetraplegia) belong to the most severe locomotor disabilities, and functional consequences of an injury largely limit physical activity of people with tetraplegia (Morgulec and Kosmol, 2007). People with such disability require specialized rehabilitation, most often lifelong, and involvement in sports perfectly supports this process (Tasiemski and Osińska, 2013). Wheelchair rugby (WR) has been addressed mainly for people with tetraplegia who previously had no chance to participate in team sports requiring an intense physical effort (Morgulec and Skrzypczyk, 2003; Lewandowski et al., 2013). Currently WR can be also practiced by individuals with other locomotor dysfunctions satisfying current criteria of functional classification (Malone et al., 2011).

Although previous research has dealt with various aspects of practicing WR, still some areas need to be explored (Malone et al., 2011). Most previous studies have concentrated on physiological benefits related to this form of physical activity (Bhambhani, 2011), the relationship between functional ability of WR players and the classification system (Molik et al., 2008) and biomechanical analysis of wheelchair user skills (Rice et al., 2011). The results of these studies suggest that practicing WR exerts a beneficial effect on cardiorespiratory performance (Barfield et al., 2010; Moreno et al., 2013; Morgulec and Kosmol, 2007), prevents cardiovascular diseases (Abel et al., 2008), improves motor function in people with tetraplegia (Berzen and Hutzler, 2012; Lewandowski et al., 2013) and has some positive psychosocial impact (Lindemann and Cherney, 2008; Litchke et al., 2012).

While the beneficial effects of WR in the psychomotor and physiological sphere of physically disabled individuals have been a subject of extensive research, still little is known on the health and fitness risks related to this sport discipline (Fagher and Lexell, 2014; Malone et al., 2011). The problem of injuries in Paralympic sports is difficult to recognize for various reasons. These include limited interpretative possibilities of existing data due to small sample sizes, limited methods of measurement (mostly subjective methods, such as questionnaires and retrospective data collection) and short periods of evaluation (Molik and Marszałek, 2013; Willick and Webborn, 2011). Previous studies have showed that the incidence of injuries among able-bodied and disabled athletes is similar (Ferrara and Peterson, 2000), but these two groups differ in terms of types and mechanisms of experienced contusions. Two main models of acquiring injuries exist: due to the type of practiced sport and due to the type of disability presented by athletes who practice a given sport discipline (Willick and Webborn, 2011).

In this context, special attention should be paid to the sport disciplines that require the use of specialized sports wheelchairs, e.g. WR. On the one hand, WR players may be exposed to an overload resulting from everyday mobility in a wheelchair and activities related to its use. On the other hand, specific nature of WR may also play an important role. WR is a dynamic and contact sport discipline (e.g. intentional collisions between wheelchairs are permitted), which potentially increases the risk of sports injuries among players (Vanlandewijck et al., 2001). Moreover, athletes who practice WR use training methods typical for able-bodied athletes. Such an approach with no doubt results in an increase in their sports level, but is also associated with greater injury risk (Molik and Marszałek, 2013).

Sports injuries can be defined as bodily injuries that arise during training or competition, and stopped, limited or modified participation in sports activities for one day or more (Ferrara and Buckley, 1996; Kegel and Malchow, 1994). Such a definition of sports injuries has been used in previous studies (Burnham et al., 1994; Taylor and Williams, 1995). Negative effects of sports injuries (e.g. injury of the upper limb) experienced by individuals with tetraplegia are much broader than in able-bodied athletes. Aside from a discontinuation of sporting activities, such injuries may hinder or even preclude basic activities of daily living, such as dressing, undressing, washing, propelling the wheelchair, etc.

Therefore, a post-traumatic musculoskeletal dysfunction has much more serious consequences in physically disabled athletes than in able-bodied subjects (Willick and Webborn, 2011). Aside from the need for treatment and discontinuation of training, sports injuries mean radical changes in everyday functioning of individuals with tetraplegia, often making them dependent on other people. This emphasizes the need for characterization of the injuries (a type and a location, a situation in the game which caused an injury, etc.) experienced by players who use wheelchairs. Accurate medical diagnosis and identification of risk factors for WR-related injuries constitute the basis for preventive measures. Relevant knowledge on the WR-related injuries is a prerequisite of recommendations for coaches and players, aimed at elimination or at least minimization of related adverse effects (Molik and Marszałek 2013).

Previous studies dealing with injuries in Paralympic athletes have showed that they typically experience minor contusions that result in up to 7-day discontinuation of training and do not require a long-term convalescence (Molik and Marszałek, 2013). Previous research on sports injuries experienced by physically disabled team sport players included representatives of such disciplines as wheelchair basketball (Danis and Mikuła, 1999), sitting volleyball (Wieczorek et al., 2007) and volleyball (Bolach et al., 2010). The authors of these studies have reported that such sports are associated with injuries resulting from excessive strain, rather than with acute injuries, and emphasized the need to further improve injury prevention. Moreover, they have emphasized the need to determine the etiology of injuries emerging in Paralympic sports (Molik and Marszałek, 2013). Other researchers pointed to an important role of a relationship between aggressiveness in sports and the incidence of injuries (Visek et al., 2010).

The aim of this study was to analyze the incidence of sports injuries among WR players. Moreover, we verified if the levels of aggressiveness and anger presented by the athletes and their roles in the team (defense/offence) influenced the incidence and severity (requiring/non-requiring a medical intervention) of injuries. We assumed that offensive players with higher levels of aggressiveness and anger were exposed to more frequent and more severe injuries than defensive players characterized by lower levels of aggressiveness and anger.

## Material and Methods

### Participants and Procedures

The study included 24 men, members of the Polish National WR Team, among them 14 players from the A and B squads, and 10 players aspiring to join the main squad, defined as C squad. During a 9-month observation period (between March and November 2014), the athletes participated in up to 9 training camps and 4 WR tournaments. The mean number of training camp and tournament days was 18 (SD=10.13) with the range between 4 and 36 days. Due to such considerable heterogeneity, we decided to analyze solely the players who participated in training camps and tournaments for at least 18 days.

A total of 14 WR players (12 from squad A/B and 2 from squad C), with a mean number of 25 training and tournament days (SD=5.68), were eventually subjected to the analysis. All training sessions and tournaments started with a 30 min warm-up and ended with a 15 min stretching routine. All injuries that did not require a medical intervention were properly and timely classified by National Team physiotherapists, among them the first author of this paper. The data of injuries that needed to be consulted by a physician were extracted from medical histories of the athletes.

### Measures

The study was based on the following instruments:

1. The Competitive Aggressiveness and Anger Scale (CAAS), designed to identify athletes prone to exhibit acts of aggression and anger (Maxwell and Moores, 2007). The questionnaire consists of 12 questions: 6 evaluating the level of aggressiveness, and another 6 evaluating the level of anger. The elements of aggressiveness refer to the willingness to use physical and verbal abuse. The elements of anger describe the degree or level of irritation resulting from a loss, and the degree or level of negative emotions directed at opponents. The answers are given on a 5-item Likert-type scale, with 1 corresponding to “almost never” and 5 to “almost always”. The higher the score, the higher the levels of aggressiveness and anger presented by a given athlete. Maxwell and Moores (2007) confirmed high reliability of the CAAS (Aggressiveness: α=0.83–0.84; Anger: α=0.78–0.83) in a group of able-bodied rugby players.
2. A register of sports injuries that did not require a medical intervention. Respondents provided information about their injuries (a type and a location, duration of resultant training absence) and circumstances of their occurrence (place, the position in the game, the match or training phase and the type of strain which resulted in the injury).
3. A register of sports injuries that required a medical intervention, providing information about the injury: date and circumstances, date of medical intervention, diagnostic tests performed, the diagnosis, the type and duration of treatment, duration of training absence, duration of symptoms.
4. A demographic questionnaire to gather personal data, such as the date of birth, sex, the educational level, occupation, the type of disability, the date of injury or the diagnosis of disease and sports experience.

The group of 14 players included 9 high-pointers, i.e. offensive players (2.0–3.5 pts.), and 5 low-pointers, i.e. defensive players (0.5–1.5 pts.). Mean age of the players was 29.5 years (SD=5.71). Their mean experience in WR was 6.68 years (SD=3.66), and the mean amount of time spent on rugby training amounted to 377 min per week (SD=142). Twelve players had a cervical SCI, usually due to a diving accident (n=8). Most athletes lived in an urban setting (n=12) and were involved in tertiary studies at the time of the research (n=11). Detailed demographic characteristics of the study participants are presented in Table 1.

### Statistical analysis

All statistical analyses were conducted with IMB SPSS Statistics 21. Descriptive statistics (frequencies, means, standard deviations) were used to present basic demographic data. The Mann-Whitney U-test was conducted to compare the incidence of sports injuries and the levels of anger and aggressiveness presented by offensive and defensive players. The relationship between the levels of anger and aggressiveness and the incidence of sports injuries was analyzed on the basis of the Spearman rank correlation coefficient (r_s_). The incidence rate for sports injuries was computed as the number of injuries divided by the number of players and the mean number of training and tournament days (Magno e Silva et al., 2013).

## Results

During the analyzed follow-up period, Polish WR National Team players sustained a total of 102 injuries that did not require a medical intervention, namely muscle strains (n=19), muscle overloads (n=43), abrasions (n=30), subluxations (n=2) and bruises (n=8), as well as four injuries that needed to be consulted by a physician: a multi-joint spinal overload due to degenerative changes, a strain of the supraspinatus muscle (proximal insertion), bruised ribs (right side) and olecranon bursitis. The incidence of injuries corresponded to 0.3 per player per training day. Two injuries that required a medical intervention, the muscle strain and bruised ribs, occurred after a player fell down on the floor during the game (after hitting the opponent’s wheelchair), and another two resulted from degenerative changes in the spine and multiple muscle overload, respectively. One of the injuries that was not consulted by a physician (bruised ribs) required a 24-hour training absence, and one injury being a subject of medical intervention (strain of the supraspinatus muscle) excluded the player from training for 2 months.

Detailed characteristics of the injuries experienced by players from the Polish WR National Team are presented in Table 2. As shown in the table, defensive and offensive players differed in terms of the incidence of injuries. During the analyzed follow-up period, three offensive players sustained 15 or more injuries, and a considerable proportion of athletes from this group experienced one, two or three injuries. Defensive players showed lesser variance in the incidence of sports injuries (range: 3–8).

Shoulder girdle muscles (n=28) and arm muscles (n=18) were the most common locations of the injuries that were not consulted by a physician (Table 3). Shoulder girdle muscles were typically overused (n=22). Abrasions were located mainly on the sides of the trunk (n=17). Muscle strains were the most common type of injury to arm (n=7) and shoulder girdle muscles (n=6). Subluxations (n=2) and bruises (n=3) typically involved hand joints and muscles.

Although offensive players experienced the injuries that were not consulted by a physician more frequently (mean=8.22; SD=6.26) than defensive players (mean=5.60; SD=1.82), this difference was not statistically significant (U=25.500; p=0.699). All four injuries that required a medical intervention (Table 2) were reported by offensive players.

Offensive players had a higher mean level of anger and aggressiveness in sports (CAAS=81.68; SD=11.91) than defensive players (CAAS=73.01; SD=8.61), but also this intergroup difference turned out to be non-significant (U=33.000; p=0.190). Moreover, no significant association was found between the levels of anger and aggressiveness and the incidence of sports injuries that did not require a medical intervention (rs=0.024; p=0.934).

## Discussion

The aim of this study was to analyze sports injuries (both requiring and non-requiring a medical intervention) sustained by WR players (members of the Polish National Team and athletes aspiring to the team) during a 9-month period of training and competition. We determined the incidence and severity of the injuries and verified if these two parameters were associated with the levels of aggression and anger presented by the players and their roles in the team (defense/offence). Over the 9-month period, 14 WR players sustained a total of 106 injuries (both requiring and non-requiring a medical intervention). This corresponded to 0.3 injury per athlete per training day; exactly the same incidence rate of sports injuries was previously reported by Magno e Silva et al. (2013) in a group of visually impaired swimmers.

According to Ferrara and Peterson (2000), WR should be classified among disciplines with a high risk of injury. Such a classification is supported by the data included in the report on the incidence of injuries among participants of the London 2012 Summer Paralympic Games, in which WR ranked as the fifth most injury-prone discipline (Willick et al., 2013). Surprisingly, a Polish study carried out by Molik et al. (2011) did not identify WR as an injury-predisposing sport. However, the results of this latter study are also inconsistent with our hereby presented findings pointing to a relatively high incidence of injuries among WR players. Similarly to previous studies, we showed that most contusions experienced by disabled athletes were minor injuries without serious medical consequences (Ferrara and Peterson, 2000; Molik et al., 2011). Up to 96% of the injuries identified in our research did not require a medical intervention and only 4% were consulted by a physician. Furthermore, only two of the hereby reported injuries resulted in a temporary inability to participate in training sessions.

On the basis of the literature review, Ferrara and Peterson (2000) identified a relationship between the location of injury, the type of disability and a practiced sports discipline. Athletes practicing wheelchair sports were shown to be more prone to injuries of upper extremities (Klenck and Gebke, 2007; Willick et al., 2013). Our findings are consistent with this data as upper extremity injuries represented up to 63% of all contusions reported by WR players. Detailed analysis revealed that 42% of all injuries were strains (34% of upper extremity strains and remaining 8% of strains involving muscles of the spine and joints of the head). This likely resulted from a cumulative strain of the upper extremity related to both practicing WR and activities of daily living that require moving about on a wheelchair. A similar phenomenon was previously reported by Finley and Rodgers (2004) who analyzed the incidence of shoulder injuries among wheelchair users (both athletes and non-athletes). They showed that involvement in sports neither increased nor decreased the risk of shoulder joint pain incidents. Therefore, at least some of the injuries experienced by our athletes might be related to their activities of daily living. This hypothesis is supported by the results of previous studies, in which some activities of daily living, such as propelling the wheelchair, transferring from seat to seat and reaching above the head, turned out to be linked to pain in the shoulder joint area (Subbarao et al., 1995). Consequently, individuals using wheelchairs on a daily basis should be encouraged to undertake regular physical activity. On the other hand, at least part of the injuries experienced by wheelchair users are with no doubt related to their involvement in the training process. Training overload and resultant accumulation of microinjuries in soft tissues of the upper extremities are probably a key risk factor of injury. Noticeably, the studies mentioned above included individuals who practiced sports with various intensities (both at a recreational and competitive level), and were not controlled for training loads. Consequently, no firm conclusions can be formulated, and the questions to what extent the cumulative strain contributes to sports injuries in disabled athletes and how it is affected by loads inherent to the activities of daily living should be addressed in future research.

In our study, strains were more prevalent among defensive players (50% of all injuries noted in this group) than in offensive players (39%). Perhaps, higher risk of the cumulative strain (related to both training and activities of daily living) and resultant injury reflected a greater extent of disability in defensive players and a lower regenerative potential of their functionally weaker muscles. We showed that the incidence and type of injury varied depending on the players’ role (defense/offence). The incidence of injuries among offensive players was higher than in defensive players. Although the intergroup difference was not statistically significant, this tendency should be considered in recommendations for WR coaches, especially that also Molik et al. (2011) showed that the players who were more fit (represented a higher sports class), and as such had more offensive roles, sustained injuries more often. Interestingly, the same researchers found an inverse relationship in wheelchair basketball players, namely more injuries were noted among less functionally able subjects. Consequently, in WR, in which the division into offence and defense players is clear, playing in attack (which is associated with a higher level of functional fitness) seems to be a risk factor of injury.

To find a reason behind the different incidence of injuries among defense and offence WR players, we compared their levels of anger and aggressiveness in sports. Not surprisingly, offensive players showed a tendency to higher levels of anger and aggressiveness than defensive players. However, we did not find a significant correlation between the level of anger and aggressiveness and the incidence of sports injuries among WR players.

Our findings imply that coaching staff of the Polish WR National Team are aware of the injury risk inherent to this discipline and apply all respective preventive measures (Molik et al., 2011; Molik et al., 2013), such as a routine 30 min warmup before the workout and 15 min stretching at the end. Only 6% of all the injuries reported by our athletes were hand abrasions, which probably reflects appropriate use of necessary protective equipment, especially rubber gloves that protect players’ hands from abrasions. Furthermore, only 2% of the abrasions and bruises involved lower limbs, which probably resulted from the fact that WR players used special sport wheelchairs with bumpers protecting them from abrasions and bruises of lower limbs. Nevertheless, despite the use of all these protective measures, a significant proportion of injuries (25%) were abrasions and bruises of the torso, abdomen and back. Consequently, coaches and medical staff taking care of WR players should consider protection of these body areas, e.g. with special trunk protectors, in order to reduce the injury risk.

Since sports injuries have serious consequences for both athletes (training absence, difficulties in independent living) and society (medical expenses, absenteeism at school or work), the issue is one of the leading research topics recommended by the International Paralympic Committee. However, we still lack reliable research results in this area, especially with regard to WR (Fagher and Lexell, 2014). The findings of the present study partially fill this gap. One strength of our research stems from the fact that it was not a retrospective analysis. All data were collected prospectively, and each case of injury was examined and recorded by a qualified physiotherapist, using a dedicated card. The data collected in such a structured manner provided a basis for research on the etiology of injuries experienced by WR players. We are well aware that a relatively small sample size limits the possibility of generalizing our findings on the whole population of WR players. It should be noted, however, that reduction of the sample size to 14 WR players provided greater homogeneity of the group. The group of athletes who were initially enrolled to the study varied considerably in terms of the number of training and tournament days, and therefore, we limited the sample to those WR players who actively participated in the training process.

The low turnout of disabled athletes in training/competition is a commonly observed phenomenon in Poland. This reflects some particularities of the Paralympic sport organization system in the country, primarily resulting from insufficient funding. Lack of financial resources prevents many eager players from participating in all training camps and tournaments. Therefore, to keep the European level of the game, Polish WR National Team players need to maintain a high form, training both in their clubs and at home. It should be noted that also the shortage of proper equipment is a consequence of insufficient funding. Modern wheelchairs dedicated to WR are designed with particular emphasis on the safety and comfort of the players, which should be reflected by a lower incidence of injuries. However, the better equipment is more costly and lack of sufficient financial resources enforces the use of wheelchairs of poorer quality and standard.

## Conclusions

The following observations were made during the 9-month period corresponding to a mean of 25 training and tournament days:

1. Polish WR National Team players experienced primarily minor sports injuries (n=102), such as muscle overload and strains, abrasions in the upper limbs and trunk, that did not require a medical intervention.
2. Only four injuries required a medical intervention. Two of them occurred after a player fell down on the floor during the game (after collision with the opponent’s wheelchair), and another two resulted from degenerative changes in the spine and multiple muscle overload, respectively.
3. Sports injuries occurred more frequently among offensive than defensive players, but this difference was not statistically significant.
4. Offensive players showed a tendency to higher levels of anger and aggressiveness than defensive players, but no significant correlation was found between the latter variable and the incidence of sports injuries.

## Figures and Tables

**Table 1:** Demographic characteristics of Polish WR National Team players

Polish WR National Team Players (n=14)	
*Type of disability*	
SCI, C5 level	3
SCI, C6 level	8
SCI, C7 level	1
Other	2
*Disability cause*	
Road traffic accident	3
Diving accident	8
Sports accident	1
Other reason	2
*Residence*	
Urban setting	12
Rural setting	2
*Occupational status*	
Student	2
Employed	11
Unemployed	1

**Table 2:** Detailed characteristics of injuries that did not require a medical intervention, experienced by Polish WR National Team players (n = 14)

		Injuries non-consulted by a physician					
			
No.	Player	strains	overloads	abrasions	subluxations	bruises	Total
1*	Offensive	3	6	6	0	2	17
2	Offensive	3	8	4	0	1	16
3	Offensive	1	6	6	0	2	15
4*	Offensive	1	6	0	0	1	8
5*	Offensive	1	3	2	1	0	7
6	Offensive	2	0	3	0	0	5
7	Offensive	0	0	2	0	1	3
8*	Offensive	0	0	1	1	0	2
9	Offensive	1	0	0	0	0	1

	Total (n) 1	12	29	24	2	7	74
	Total (%) 1	16%	39%	32%	3%	9%	100%

1	Defensive	2	3	3	0	0	8
2	Defensive	3	1	1	0	1	6
3	Defensive	2	4	0	0	0	6
4	Defensive	0	5	0	0	0	5
5	Defensive	0	1	2	0	0	3

	Total (n) 2	7	14	6	0	1	28
	Total (%) 2	25%	50%	21%	0	4%	100%

In the study group (n)		19	43	30	2	8	102
In the study group (%)		19%	42%	29%	2%	8%	100%

* additionally an injury consulted by a physician;

1 offensive players;

2 defensive players

**Table 3:** Muscle groups/body areas involved in injuries that were not consulted by a physician (n; % of total injuries)

Muscle groups/body areas involved in injuries non-consulted by a physician				

Strains	overloads	abrasions	subluxations	Bruises
SGM (22; 21.6%)	ER (1; 1%)	SGM (6; 5.9%)	HJM (2; 2%)	ER (1; 1%)
AM (11; 10.8%)	HJM (6; 5.9%)	AM (7; 6.9%)		HJM (3; 2.9%)
ER (2; 2%)	ST (17; 16.7%)	HJM (3; 2.9%)		LLM (2; 2%)
MHN (8; 7.8%)	AB (1; 1%)	LLM (3; 2.9%)		Ribs (2;2%)
	B (5; 4.9%)			

(43; 42.2%)	(30; 29.4%)	(19; 18.6%)	(2; 2%)	(8; 7.8%)

SGM- shoulder girdle muscle, MHN- muscles of head and neck, HJM- hand joint muscles, ST- sides of the trunk, LLM- lower limb muscles, AM- arm muscles, ER- elbow region, AB- abdomen, B- back, R- ribs
